# Lithium enhances survival and regrowth of spinal motoneurons after ventral root avulsion

**DOI:** 10.1186/1471-2202-15-84

**Published:** 2014-07-02

**Authors:** Rao Fu, Ying Tang, Ze-Min Ling, Ying-Qin Li, Xiao Cheng, Fa-Huan Song, Li-Hua Zhou, Wutian Wu

**Affiliations:** 1 Department of Anatomy, Zhongshan School of Medicine, Sun Yat-sen University, No. 74, Zhongshan Road 2, Guangzhou, China; 2 Department of Anatomy, Li Ka Sheng Faculty of Medicine, The University of Hong Kong, Hong Kong, SAR, China; 3 State Key Laboratory of Brain and Cognitive Sciences, The University of Hong Kong, Hong Kong, SAR, China; 4 GHM Institute of CNS regeneration, Jinan University, Guangzhou, China

**Keywords:** Lithium, Reimplantation, Motoneuron, Axonal regeneration, Brachial root avulsion

## Abstract

**Background:**

During the clinical treatment of the brachial plexus root avulsion (BPRA), reimplantation surgery can not completely repair the motor function of the hand because the axonal growth velocity of the spinal motoneurons (MNs) is too slow to re-innervate the intrinsic hand muscles before muscle atrophy. Here, we investigated whether lithium can enhance the regenerative capacity of the spinal MNs in a rat model of BPRA.

**Results:**

The avulsion and immediate reimplantation of the C7 and C8 ventral roots were performed and followed with daily intraperitoneal administration of a therapeutic concentrationof LiCl. After a 20 week long-term rehabilitation, the motor function recovery of the injured forepaw was studied by a grasping test. The survival and regeneration of MNs were checked by choline acetyltransferase (ChAT) immunofluorescence and by Fluoro-Gold (FG) retrograde labeling through the median and ulnar nerves of the ventral horn MNs. The number and diameter of the nerve fibers in the median nerve were assessed by toluidine blue staining. Our results showed that lithium plus reimplantation therapy resulted in a significantly higher grasping strength of the digits of the injured forepaw. Lithium plus reimplantation allowed 45.1% ± 8.11% of ChAT-positive MNs to survive the injury and increased the number and diameter of nerve fibers in the median nerve. The number of FG-labeled regenerative MNs was significantly elevated in all of the reimplantation animals. Our present data proved that lithium can enhance the regenerative capacity of spinal MNs.

**Conclusions:**

These results suggest that immediate administration of lithium could be used to assist reimplantation surgery in repairing BPRA injuries in clinical treatment.

## Background

Ventral root avulsion is not similar to distal axotomy, which does not cause MN death in adult animals [1-3]. In contrast, avulsion of the spinal roots isolates the MNs from peripheral nerves and glial cells, causing several interrelated damage processes in MNs, including morphological alterations, biochemical disturbances, gene expression dysregulation, metabolic changes and cell deathin the affected spinal cordsegments [1,4-8]. The vast majority of the corresponding spinal MNs die within 2–6 weeks of injury in adult rats [9,10], resulting in paralysis of the corresponding muscle groups because the brachial plexus is the only nerve supply to the upper limb. Surgical reimplantation of avulsed ventral roots can rescue the MNs in this model. However, the implantation, which inserts the avulsed ventral rootlets into the parenchyma of the spinal cord, may cause additional damage to the spinal cord and requires more challenging surgical skills. In this study, we employed a new microsurgical technique to restore the connection by positioning the avulsed ventral root on the ventrolateral pial surface of the spinal cord instead of inserting the ventral rootlets into the parenchyma of the spinal cord [4,11,12]. However, the growth velocity of the regenerative MN axons is too slow to re-innervate the intrinsic forepaw muscles before the denervated muscle atrophies [13,14]. We also estimated the effect of drugs on the enhancement of the growth velocity of the regenerative MN axons. Lithium, which is extensively used in the treatment of bipolar mood disorder in clinical settings, has been demonstrated to be neuroprotective against a variety of neuronal insults, such as amyotrophic lateral sclerosis [15-17]. Moreover, lithium has been demonstrated to promote axon regeneration [18]. Our previous study also found that lithium can reinforce the axonal regeneration of rubrospinal tract neurons in chondroitinase ABC treated rats after spinal cord hemisection [19,20]. However, whether lithium can promote the regeneration of the spinal MNs after ventral root avulsion is still unknown. Here, we tested the effects of therapeutic lithium on the regeneration of spinal MNs of BPRA-injured and reimplantation-treated adult rats.

## Results

### Lithium and reimplantation effects on the motor functional recovery of the injured forepaw

**Figure 1:**
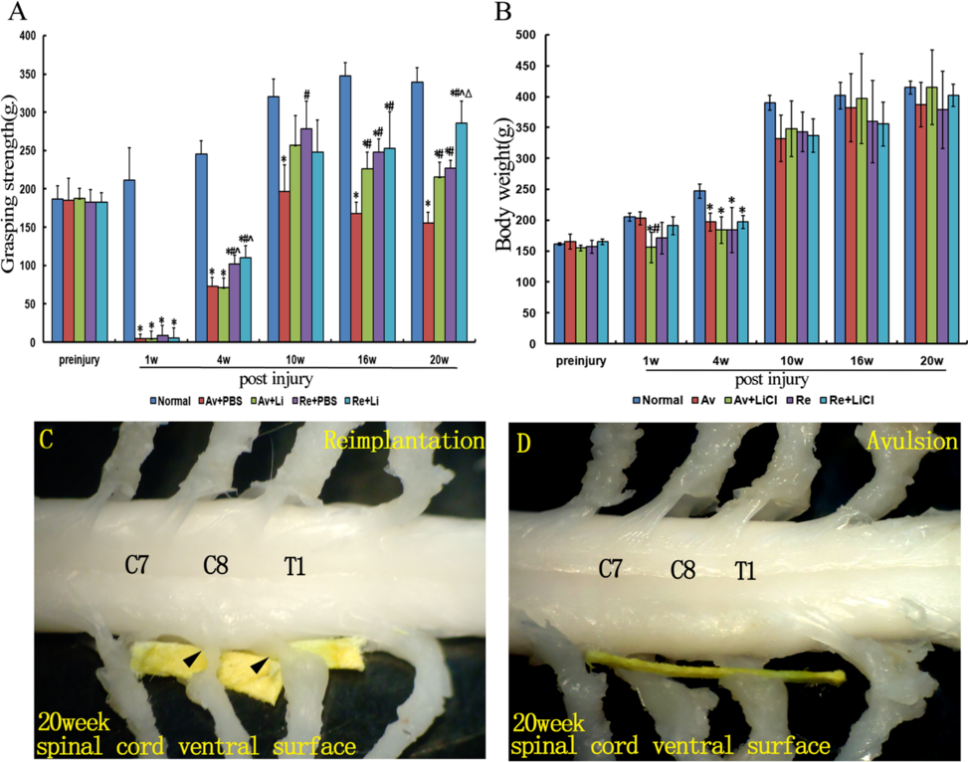
**Performance of rats in the grasping test and the gross anatomy of the cervical spinal cord after C7 and C8 ventral root avulsion.** Figure 1A shows the average grasping strength of the ipsilateral forepaws in each subgroup of rats at each timepoint before and after the operation and therapies **(A)**. Figure 1B shows the average body weights in each subgroup at each timepoint before and after the operation and therapies. *p < 0.05compared to the normal control group, #p < 0.05 compared to the Av + PBS subgroup, ^p < 0.05 compared to the Av + Li subgroup, and Δp < 0.05 compared to the Re + PBS subgroup **(B)**. Figure 1C and D show the photographs of the gross anatomy of the cervical spinal cord of reimplantation **(C)** and avulsion **(D)** animals at the end of the 20 week postinjury period. **(C)** For reimplantation rats, a nerve was found that was attached close to the ventrolateral surface of the ipsilateral C7 spinal segment and regrown in the middle the trunk of the ipsilateral brachial plexus (arrows). Additionally, the other reimplanted ventral root that originated from the ventral surface of the ipsilateral C8 spinal segmentwas identified regrowing into the lower trunk of the ipsilateral brachial plexus (arrows). **(D)** An obvious gap between distal part of the C7 and C8 spinal nerves and the ventral surface of the correspondingspinal cord was detected in avulsion animals (yellow stick).

The grasping test was applied to the ipsilateral (right side) forepaws of all of the experimental rats at three days pre-lesion and from 1 to 20 weeks post-lesion. At 3 days pre-lesion, there was no significant difference in the grasping strength among all of the 5 subgroups (p > 0.05, Figure 1A). At the first week post-lesion, avulsion of the C7 and C8 ventral roots resulted in almost a total loss of the grasping strength in both the Re and Av subgroups when compared to the normal control subgroup (all p < 0.05, Figure 1A), moreover, an equal loss of function occurred in all of the avulsion-injured subgroups (p > 0.05 among the 4 experimental subgroups, Figure 1A). One month later, a significant increase of the grasping strength occurred accompanied by an increase in body weight in each subgroup (Figure 1A,B). Among the different subgroups of the rats at 4 weeks post-lesion, significant improvements in grasp strength were shown in both Re subgroups when compared to those of both Av subgroups (all p < 0.05, Figure 1A). However, the average grasping strengths were still far weaker in both Re subgroups compared to that of the normal subgroup (all p < 0.05, Figure 1A). An obvious recovery of the grasping strength was demonstrated on evaluation from 10 weeks post-lesion in the Av + Li, Re + PBS, and Re + Li subgroups, and the levels of the grasping strength in these 3 subgroups were similar to and not significantly different from that of the normal subgroup (all p > 0.05, Figure 1A). At 10 weeks post-lesion, the grasping strength in the Av + PBS subgroup was significantly lower than those of the Re + Li and normal subgroups (all p < 0.05, Figure 1A). From 16 to 20 weeks post-lesion, the grasping strength levels of the Re + Li, Re + PBS, and Av + Li subgroups were all significantly lower than that of the normal subgroup (all p < 0.05, Figure 1A), but significantly higher than that of the Av + PBS subgroup (all p < 0.05, Figure 1A). At the end of the study, an obvious enhancement of the grasping strength appeared in the Re + Li subgroup, which was significantly higher than those of both the Re + PBS and Av + Li subgroups (all p < 0.05, Figure 1A). The most interesting result was that the grasping strength was not significantly different between the Re + PBS and Av + Li subgroups from 10–20 weeks post-lesion (all p > 0.05, Figure 1A).

### Gross anatomical observation

After the behavioral test, rats were euthanized, and then the spinal cord was carefully dissected to ensure that the C7 and C8 ventral roots had been completely avulsed or successfully re-implanted into the spinal cord. Under a surgical microscope, we found 2–3 intact ventral rootlets of spinal nerves attached to the spinal cord at the anterolateral sulcus in the contralateral C7 or C8 spinal segments (Figure 1C-D). For ventral root reimplantation treated rats, a nerve root was found that was closely attached to the ventrolateral surface of the ipsilateral C7 spinal segment and regrew into the middle trunk of the ipsilateral brachial plexus. Additionally, the other reimplanted ventral root was identified as a nerve that originated from the ventral surface of the ipsilateral C8 spinal segment, and regenerated into the lower trunk of the ipsilateral brachial plexus (Figure 1C). Conversely, for the rats without ventral root reimplantation treatments, there was an obvious gap between the C7 and C8 spinal segments and the trunks of the brachial plexus in avulsion rats (Figure 1D). In all animals of the present study, the C7 and C8 dorsal roots were well connected to the dorsal aspect of the C7 and C8 spinal segments, respectively.

### Lithium and reimplantation enhanced the survival of injured motoneurons

**Figure 2:**
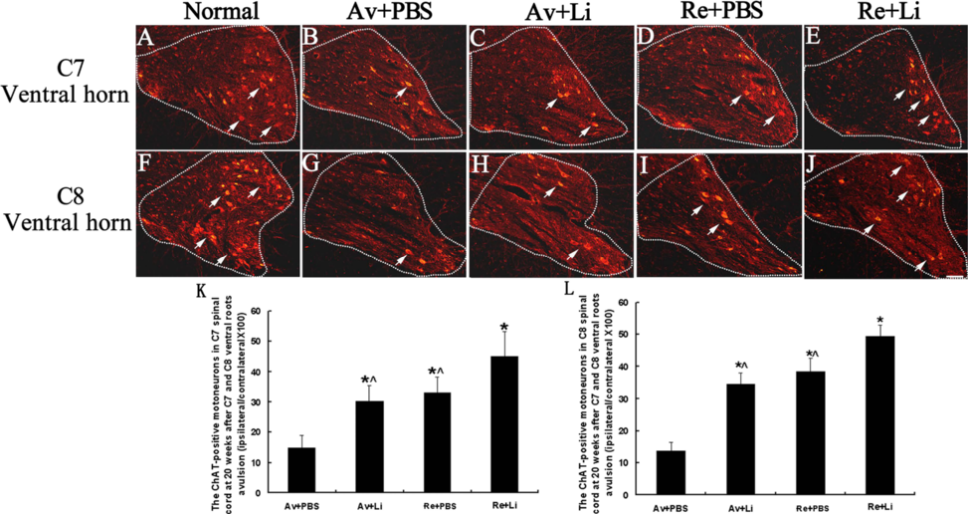
**Effects of ventral root reimplantation and lithium on the survival of avulsedmotoneurons as revealed by the ChAT-immunofluorescence reaction at 20 weeks post-reimplantation.** Representative micrographs of ipsilateral C7 **(A-E)** and C8 **(F-J)** spinal cross sections showing ChAT-positive motoneurons present in the ventral horn of animals in the normal control **(A, F)**, Av + PBS **(B, G)**, Av + Li **(C, H)**, Re + PBS **(D, I)**, and Re + Li **(E, J)** treated subgroups. The MNs showing a cytoplasm stained with the ChAT antibody and a visible nucleus were counted (arrows) on both sides of each C7 and C8 ventral horn (the outline) under a 20× objective lens. Scale bar = 100 μm. **(K-L)**: The percentage of the number of ChAT-positive motoneurons in the C7 (K) and C8 (L) ventral horns (ipsilateral normalized to contralateral) was compared among all the subgroups. Lithium alone saved some of the affected motoneurons. Lithium plus reimplantation resulted in significantly more of the affected motoneurons surviving the avulsion injury compared to those that underwent single lithium or single reimplantation surgery. *p < 0.05, compared to the Av + PBS subgroup, ^p < 0.05 compared to the Re + Li subgroup.

The survival of the injured MNs was assessed by quantifying the number of ChAT-positive MNs at 20 weeks post-lesion (Figure 2). In the ipsilateral ventral horns of the C7 spinal segments (Figure 2A-E), the number of ChAT-positive MNs (percentage of the contralateral ventral horns in the same spinal section) was only 14.9% ± 4.13% in the Av + PBS subgroup (Figure 2B), while the value increased to 30.2% ± 5.39% in the Av + Li subgroup (Figure 2C), to 33.2% ± 5.14% in the Re + PBS subgroup (Figure 2D) and to 45.1% ± 8.11% in the Re + Li (Figure 2E) subgroup. The effects of lithium and reimplantation on the survival of C8 MNs were similar to those of the C7 spinal segment (Figure 2F-J). The number of ChAT-positive MNs was lowest in the Av + PBS subgroup (13.6% ± 2.63%, Figure 2G), higher in the Av + Li (34.7% ± 3.47%, Figure 2H) and Re + PBS subgroups (38.5% ± 4.21%, Figure 2I), and highest in the Re + Li (49.3% ± 3.71%, Figure 2J) subgroup. Statistical analysis showed that the number of ChAT-positive MNs was higher in the Re + Li subgroupand, in contrast, lower in the Av + PBS subgroup when compared to any of the other treated subgroups (all p < 0.05, Figure 2K,L). The number of ChAT-positive MNs in both the C7 and C8 ipsilateral ventral horns was not significantly different between the Av + Li and Re + PBS subgroups (all P > 0.05, Figure 2K,L).

### Lithium and reimplantation promoted axon regeneration of injured motoneurons

**Figure 3:**
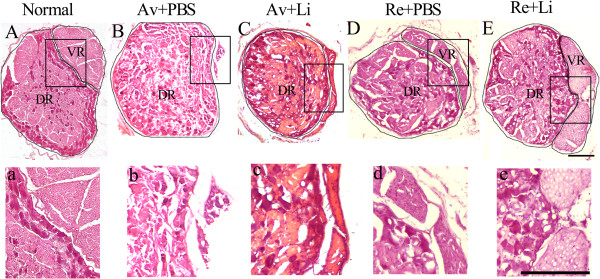
**The morphology of the dorsal root ganglion and ventral roots (cross section) with HE staining at 20 weeks after C7 and C8 ventral root avulsion.** In normal group, the boundary of the DRG and ventral root were clearly recognized. The structure of axons in the ventral roots were well organized **(A-a)**. In the Av + PBS subgroup, only the boundary of the DRG could be found, and the ventral roots were totally absent **(B-b)**. In the Av + Li subgroup, the intact structure of the DRG was demonstrated with the residual part of the avulsed ventral root **(C-c)**. In the Re + PBS subgroup, the regenerative ventral root was clearly recognized with 3 rootlets adjacent to the DRG wrapped in an intact epineurium. A large amount of Schwann cells were distributed among the axons **(D-d)**. In the Re + Li subgroup, the ventral roots were closely attached to the DRG with an intact epineurium, and some axons in the regenerative ventral roots were wrapped with a thinner myelin sheath **(E-e)**. Scale bar = 200 μm.

**Figure 4:**
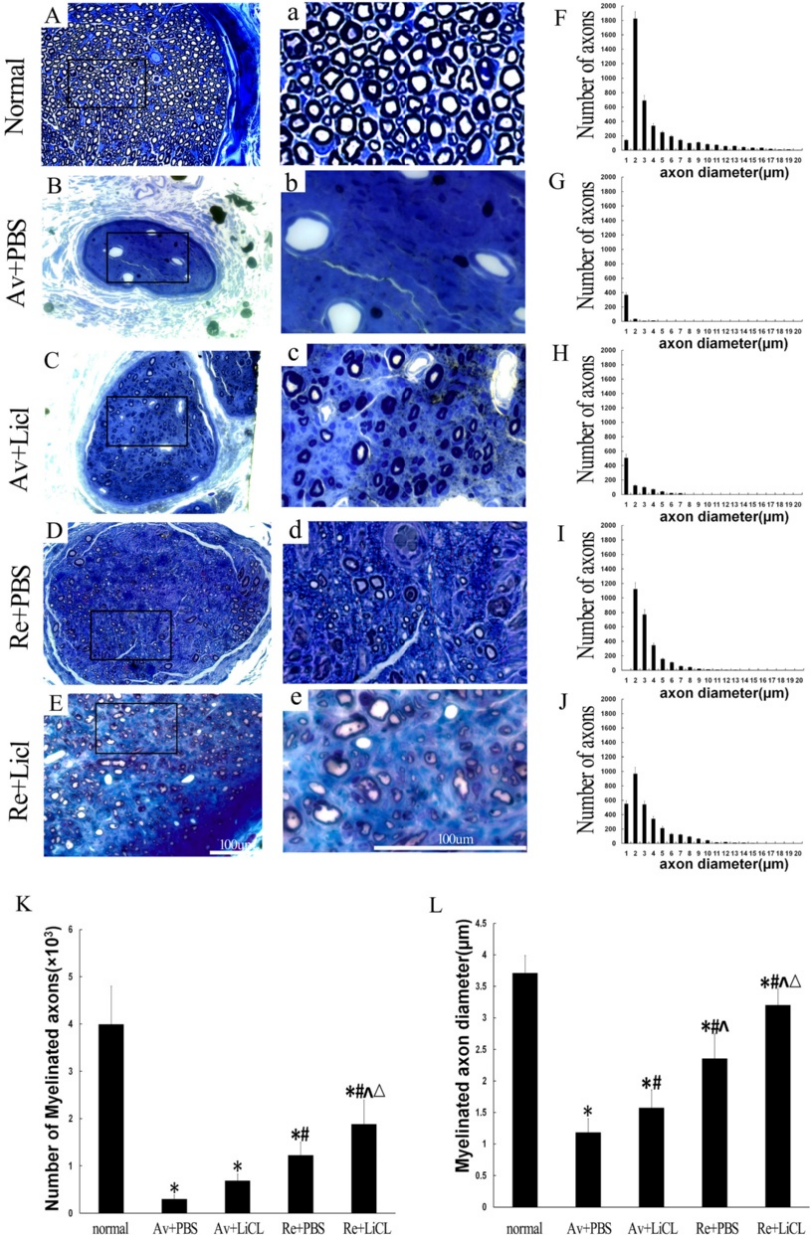
**The effects of ventral root reimplantation and lithium on the axonal regeneration in the median nerves at 40 mm distal to the reimplantation site at 20 weeks post-reimplantation.** Figures 4**A-E** and **a-e:** A large of number of myelinated axons were present in the normal median nerve **(A-a)**. However, the decreased presence of nerve fibers and obvious nerve atrophy were observed in both the Av + PBS **(B-b)** and Av + Li **(C-c)** subgroups. In both the Re + PBS **(D-d)** and Re + Li **(E-e)** subgroups, a small amount of uneven size nerve fibers re-appeared and were scattered in location. Scale bar = 100 μm. Figures 4**F-J:** The frequency distribution of myelinated fiber diameters in the normal control **(F)**, Av + PBS **(G)**, Av + Li **(H)**, Re + PBS **(I)** and Re + Li **(J)** subgroups were estimated. Figures 4**K-L:** The results of statistical analysis of the mean number **(4K)** and diameter **(4L)** of the median nerve were summarized. The results showed that the Av + PBS subgroup showed the lowest values and the Re + Li subgroup showed the highest values among the treated subgroups. *p < 0.05 compared to the normal subgroup, #p < 0.05 compared to the Av + PBS subgroup, ^p < 0.05 compared to the Av + Li subgroup, and △p < 0.05 compared to the Re + PBS subgroup.

At the end of the 20 weeks, both the dorsal and ventral roots of ipsilateral C7 and C8 spinal cord segments were removed and studied by HE staining (Figure 3A-E). In normal control rats, the ventral and dorsal roots of the C7 or C8 spinal cord segments were enveloped by thin root sheaths and completely separated (dotted line in Figure 3A,a). There were 2–4 rootlets in the normal C7 or C8 ventral root with well-organized axons within each rootlet (Figure 3A,a). In the rats in the Av + PBS (Figure 3B,b) and Av + Li (Figure 3C,c) subgroups, all of the ventral rootlets had disappeared, but the dorsal roots were unaffected. After reimplantation surgery, the ventral roots regenerated and were also completely separated from the dorsal root by the enveloping thin root sheath (Figure 3D-E). In the Re + PBS subgroup, approximately 2 ventral rootlets with regenerating axons were localized within the regenerating C7 or C8 ventral root (Figure 3D,d). In the Re + Li subgroup (Figure 3E,e), the two regenerative ventral rootlets were filled with well-organized axons, and the diameters of these axons were much larger, although the number of ventral rootlets was lower than that of the normal control.To further assess the effects of reimplantation surgery and lithium on the regrowth of regenerating axons in median nerves, toluidine blue staining was applied to semithin sections from the ipsilateral median nerve in the forepaw 40 mm distal to the reimplantation site. The result showed a large number of the myelinated axons in the normal control subgroup (Figure 4A,a), but a severe loss of myelinated axons in both the Av + PBS (Figure 4B,b) and Av + Li subgroups (Figure 4C,c). The high magnification of the median nerve in the avulsion series showed few myelinated fibers, with an abundance of cell debris and Schwann cells (Figure 4b,c). However, we found many small myelinated axons in the median nerve in the Re + PBS (Figure 4D,d) and Re + Li (Figure 4E,e) subgroups. Furthermore, we examined the frequency distribution of the diameter of the myelinated axons in the median nerve. In the normal controlgroup, the axon diameter distribution demonstrated a bell shape form with a left dissymmetric distribution indicating a predominance of nerve fibers with small diameters at the median nerve (Figure 4F). In the Av + PBS (Figure 4G) and Av + Li (Figure 4H) subgroups, the curves showed a more skewed distribution shape, suggesting a severe loss of nerve fibers with larger diameter, however, the axon diameter distribution showed a slight increase in the number of nerve fibers with larger diameters in the Av + Li subgroup (Figure 4H). Conversely in the Re + PBS (Figure 4I) and Re + Li (Figure 4J) subgroups, the curves of the diameter size distribution showed higher peaks, indicating a large number of nerve fibers that covered a wider range of the larger diameters.There were 3994 ± 805 myelinated axons in the normal control group, but only 303 ± 85 in the Av + PBSand 693 ± 135 in the Av + Li subgroups. The ventral root reimplantation induced substantial myelination of axons in the median nerve. The number of myelinated fibers was increased to 1225 ± 274 in the Re + PBS and 1885 ± 505 in the Re + Li subgroups. The statistical analysis showed that the difference either between the Av + PBS and the Av + Li or between the Av + Li and the Re + PBS subgroups, was not significant (all p > 0.05). A higher number of myelinated fibers was found in the Re + Li subgroup, which was significantly higher than that of the Av + PBS, Av + Li or Re + PBS subgroups but still lower than that of the normal control group (all p < 0.05, Figure 4K). The mean diameter of the myelinated axons measured 3.7 ± 0.28 μm in the normal control group, 3.2 ± 0.26 μm in the Re + Li subgroup, 2.4 ± 0.39 μm in the Re + PBS subgroup, 1.6 ± 0.29 μm in the Av + Li subgroup, and 1.2 ± 0.22 μm in the Av + PBS subgroup. Differences between all subgroups were significant (all p < 0.05, Figure 4L).

### Reimplantation, but Not lithium, promoted motoneuron regeneration

**Figure 5:**
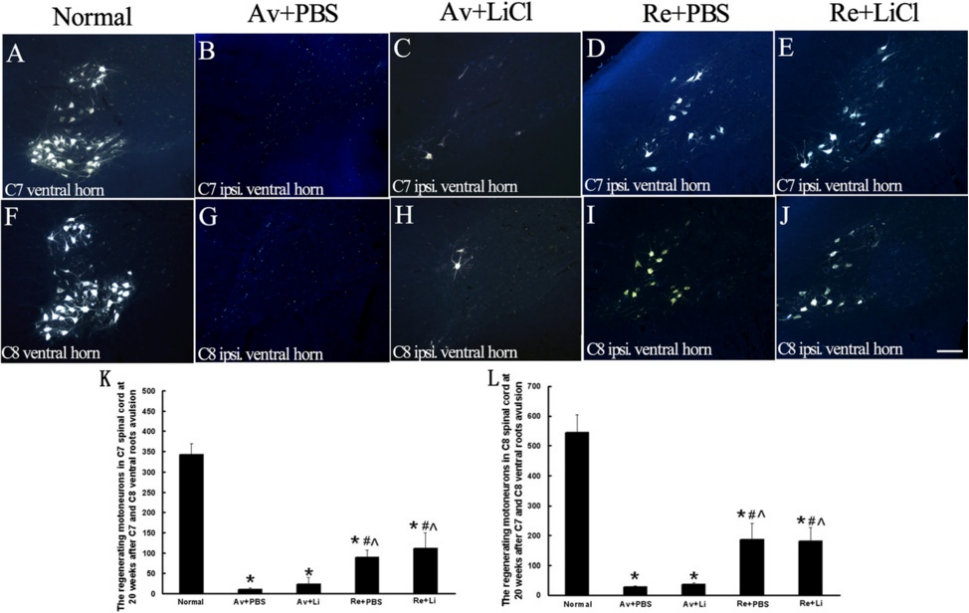
**Effects of ventral root reimplantation and lithium on the axonal regeneration of avulsed motoneurons as revealed by retrograde FG-labeling at 20 weeks post-reimplantation.** Representative micrographs of ipsilateral C7 **(A-E)** and C8 **(F-J)** spinal cross sections showing FG-labeled motoneurons present in the ipsilateral ventral horns of animals in the normal control **(A, F)**, Av + PBS **(B, G)**, Av + Li **(C, H)**, Re + PBS **(D, I)**, and Re + Li **(E, J)** subgroups. Scale bar = 100 μm. **(K-L)**: The number of regenerated motoneurons in reimplanted treated animals was significantly higher than those without reimplantation treatment both in C7 **(K)** and C8 **(L)** ventral horns. *p < 0.05 compared to the normal subgroup, #p < 0.05 compared to the Av + PBS subgroup, and ^p < 0.05 compared to the Av + Li subgroup.

At the end of 20 weeks, the Fluoro-Gold (FG) labeled MNs in the ipsilateral C7 (Figure 5A-E) and C8 (Figure 5F-J) ventral horns were counted and compared among different subgroups under a microscope. Because the FG was injected to the median and ulnar nerves at a site approximately 20 mm ± 5 mm distal to the site where the spinal cord was cut, FG-labeled MNs indicate that the retrograde tracer was taken up by the regenerating axons and transported back to the MN cytoplasm in the ipsilateral ventral horns. The quantification results showed that the number of FG-labeled MNs consisted of 329 ± 35.0 in the C7 (Figure 5A) and 545 ± 60.0 in the C8 (Figure 5F) ventral horns in the normal control subgroup. Avulsion resulted in a remarkable decrease in the number of FG-labeled MNs and consisted of 11.4 ± 2.5 in the C7 (Figure 5B) and 28 ± 3.0 in the C8 (Figure 5G) ventral horns in the Av + PBS subgroup and 29 ± 10 in the C7 (Figure 5C) and 37 ± 3.0 in the C8 (Figure 5H) ventral horns in the Av + Li subgroup. Lithium alonedid not increase the number of FG-labeled MNs because all the differences in the number of FG-labeled MNs both in the C7 and C8 ipsilateral ventral horns were not significant between the Av + Li and Av + PBS subgroups. The reimplantation surgery showed an obvious effect on the axonal regeneration of the injured MNs. The number of FG-labeled MNs was increased to 90 ± 17.4 in the C7 (Figure 5D) and 186 ± 54.9 in the C8 (Figure 5I) ventral horns in the Re + PBS subgroup, and 111 ± 38.4 in the C7 (Figure 5E) and 180 ± 45.9 in the C8 (Figure 5J) ventral horns in the Re + Li subgroup. Statistical analysis showed that the number of FG-labeled MNs was significantly higher in the Re subgroups when compared to those of the Av subgroups (all p < 0.05) in eitherthe C7 or C8 spinal segments. However, no significant difference was found either between the Av + Li and Av + PBS subgroups, or between the Re + Li and Re + PBS subgroups (all p > 0.05, Figure 5K, 5L), which indicated that lithium did not influence the number of FG-labeled MNs at 20 weeks post-lesion.

## Discussion

We show here that ventral root reimplantation can protect spinal MNs from death induced by avulsion injury and also elevate the number of regenerated MNs, as well as facilitate growth of the regenerated axon into the median nerve and promote recovery of the grasping function. In addition, systematic administration of lithium reinforces the pro-survival effect of reimplantation surgery and induces a robust increase in the number and diameter of myelinated axons in the median nerve. Moreover, we provided evidence that lithium itself can enhance the survival of injured MNs and regenerate axon regrowth in the median nerve. Here, we used the MN marker ChAT to assess the survival of the affected motor neurons. Previous studies, including ours, have reported that avulsion results in a transient absence of ChAT protein expression inside affected MNs at early post-lesion time points [21-23]. However, ChAT immunoreactivity was easily distinguished, although reduced, in the affected ipsilateral MNs in the present study. We attribute this difference to different time points in the investigation between the previous and the present studies. Previous results emphasized the early post-lesion time period (mostly within 2 weeks), but we counted the ChAT-positive MNs at the end of 20 weeks (late) post-lesion. Our result was supported by the previous study which proved that the ChAT immunoreactive intensity inside the affected lumbar MNs recovers to the normal level at 4 weeks after axonal injury [24]. Additionally, a number of previous studies, which show no significant difference in MN counts between ChAT and Nissl staining [25] or between ChAT and cresyl violet staining [26], support our results. Therefore, the number of ChAT-positive MNs can be used as a helpful marker for the survival of affected MNsatlate post-lesion times in axonal injuries of the peripheral nerves.

When repairing root-avulsion injuries, the survival of affected MNs has been confirmed to be of ultimate importance for axonal regeneration [27]. Our present data showed 33% to 40% of cervical MNs survived up to 5 months after reimplantation, which supports the previous studies that showed ventral root reimplantation is beneficial for the survival of the affected MNs [4,11,28]. The present data also support the idea that the surgically re-attached nerve acts as a conduit to induce the regenerative axons to regrow towards the peripheral target [11,13,29]. We observed a reimplanted ventral root firmly attached to the ventrolateral side of the C7 or C8 spinal cord at the proximal end connected with the ipsilateral middle or lower trunk of the brachial plexus at the distal end. In the present study, the intact C7 and C8 dorsal roots may also contribute to the repositioning of the regenerative ventral roots to the ventral root outlet area of the spinal cord and to the precise reconstruction of the ventral roots within the middle and lower trunks of the brachial plexus. According to previous studies, ventral root combined with dorsal root avulsion induces enhanced glial activation, which may occur in response to the central sensitization of nociceptive neurons at the early stage of the injury [30], and enhances glial scar formation [31,32] at the peripheral–central nervous system (PNS-CNS) transition zone at the late stage of the injury. Previously, we have also shown the avulsion induced activation of astrocytes and microglia in both white and gray matter of the injured spinal cord [23,33]. Therefore, we think that intact dorsal roots, compared to injured dorsal roots, may reduce unfavorable glial activation environments and facilitate the survival of the spinal MNs in the present study.

In addition to reimplantation itself, lithium treatment seems to positively influence the survival of affected MNs. Our data showed that the number of ChAT-positive MNs was higher in the lithium therapy group compared to the animals that only received avulsion or reimplantation. Moreover, there was no significant difference between the Av + Li subgroup and the Re + PBS subgroup in both C7 and C8 ipsilateral ventral horns. This result may imply that lithium has a neuroprotective effect by preventing neuronal death. This is in line with previous studies, for example, in primary cultures of rat cerebellar granule cells and cortical neurons, lithium robustly and potently protect against glutamate-induced, N-methyl-D-aspartate (NMDA) receptor-mediated excitotoxicity [34].

Given that the nerve fiber diameter distribution is an important method that can contribute to insights into the processes that play a role in nerve regeneration [35], the current findings suggest that reimplantation increases the number of myelinated axons and greatly ameliorates the phenotype of the lesioned nerve compared the avulsed animals. Furthermore, systemic administration of lithium may reinforce the regrowth-promoting effect of reimplantation surgery on the regenerated myelinated fibers in the median nerve. We also noted that no significant difference was found when comparing the number of myelinated axons in the Av + Li and Re + PBS subgroups, indicating that even lithium alone could protect the lesioned axon from Wallerian degeneration. This result is consistent with previous reports indicating that lithium supports regeneration in the CNS, including RGC axons [18] and rubrospinal neurons in spinal cord [19]. More importantly, a recent study reported that lithium enhances remyelination of the facial and sciatic nerves [36].

Equally interesting is our finding that a significant return of the motor function, in the form of digit flexions, in the reimplantation animals began at 4 weeks post-lesion, compared to the avulsion series. Additionally, the reimplantation plus lithium treatment group showed the highest grasp strength among all of the treated animals. This result coincided with the investigation on the spinal cord and median nerve, as well as the previous studies [37,38]. In addition, the behavioral test demonstrated that the grasping strength in avulsion injured animals treated with lithium was significantly greater than the vehicle group, and this upward trend was even maintained for 20 weeks after treatment. The rats in the two avulsion subgroups without repair surgery should not differ because there was no reimplantation to facilitate regenerating axons to grow towards the target muscle. One possible explanation for the significant effects found here could be that they result from a gradual loss of function in the Av + PBS group due to gradual muscle atrophy, which is prevented in the Av + Li group by the Li treatment. These data are consistent with a previous report that lithium induced an increase in skeletal myotube size by inhibiting GSK3β [39]. Second, anatomical dissection of the rats confirmed that the C7, C8 and T1 ventral roots make up the components of the median and ulnar nerves [40], and stimulation of the median nerve produces digit flexion in the rats [41,42]. In the present study, only the C7 and C8 ventral roots were avulsed. The ventral root of T1 was intact, which might contribute to the grasping strength that remained in the avulsed animals. The spinal MN efferent and also the somatosensory afferent from the skin and muscles of the forepaw were proven to be very important during the grasping motor tasks [43]. Our data showed that the ventral root avulsion did not affect the morphology of the corresponding dorsal roots, which indicates the intact C7 and C8 dorsal roots contributed to the somatosensory input of the forepaw and assisted in the functional recovery of digit grasping strength of the avulsed animals.

The combined therapeutic strategy of reimplantation and lithium in the present study resulted in an increase in the survival of the affected cervical MNs, an increase in the number and diameter of median nerve fibers, and finally a better recovery of motor function in the injured forepaw when compared to the single reimplantation surgery. These findingswere supported by a number of recent studies [44-47], which have shown the neuroprotective effect of lithium on neuronal trauma and degeneration [15-17]. Although some of the clinical trials show conflicting effects of the lithium ion regarding altering the progression of cognitive and functional deficits, the results share a consensus on the view that lithium may exert long-term neuroprotective effects [44,48,49]. The mechanism by which lithium confers neuroprotection has been widely studied previously. Lithium, as an inhibitor of GSK-3β, can reduce GSK-3 activity during neuronal apoptosis [50-54]. Therefore, lithium might act to reduce the oxidative stress in avulsion injured MNs in the present study because avulsion-induced oxidative stress has been demonstrated to be fatal to the affected MNs [55-57], and GSK3 has been recognized as an intermediate in oxidative stress-induced apoptotic signaling [58-60]. Another important mechanism that may contribute to lithium’s effects on the affected MNs in the present study might be attributed to lithium-induced BDNF activity in the central nervous system [61,62] because many neurotrophic factors, including BDNF, have been shown to promote survival and enhance regeneration in root-avulsion injuries [63,64]. Recently, we also found that lithium can enhance the expression of BDNF in NPCs and increases the neuronal differentiation of NPCs in the ipsilateral C7 ventral horns of the avulsion injured rats [20]. During reimplantation-induced regeneration of the MNs, production of BDNF in the distal segment of the spinal nerves and the target are also thought to account for neuronal survival and regeneration [24,65]. Therefore, lithium-induced BDNF production might also contribute to its effects onthe promotion of survival and enhancement of axonal regeneration in affected MNs in the present study. Lithium-induced activation of Bcl-2 has also been shown to suppress glial scarring and support axon regeneration in retinal ganglion cells [18,66]. As an inhibitor of GSK3, lithium induced inactivation of GSK3 was shown to promote axon regeneration and facilitate functional recovery after spinal cord transection injury [67]. Our previous study also proved that lithium can facilitate chondroitinase ABC (ChABC) to promote the axonal regeneration of rubrospinal tract neurons in spinal cord hemisection injury [19,20].

However, our data also showed that the number of FG-labeled MNs was not significantly different between the reimplantation and reimplantation plus lithium treated animals. Moreover, at a location on the median nerve 40 mm distal to the reimplantation site, the number and diameter of myelinated axons was significantly increased in the Re + Li group compared to the Re + PBS group. One possible explanation was that the good morphological structure of regenerating myelinated axons did not guarantee a 100% functional recovery. As for the goal of fully recovering paw grasping function, although we observed regenerated axons in the median nerve in the morphological study, we believe that there is a long way to go, and much more research will be required.

## Conclusions

In conclusion, systematic administration of lithium at a therapeutic dose for 20 weeks reinforcesthe pro-survival effect of reimplantation surgery on injured MNs induced by ventral root avulsion. In addition, the combined administration of lithium and reimplantation surgery promotes increases in the number and diameter of median nerve axons, as well as improves the motor functional recovery of digit flexion. The results indicate that lithium has a high potential value in surgeries that repair root-avulsion injuries in clinical settings.

## Methods

### Animals

Adult male Sprague–Dawley rats (150–180 g, Laboratory Animal Center of Sun Yat-sen University) were used in the study. The animal experiments were approved by the Committee on the Use of Live Animals for Teaching and Research of the Zhongshan School of Medicine in Sun Yat-sen University. All procedures were performed according to the Chinese National Institutes of Health Guide for the Care and Use of Laboratory Animals. A high standard of care was followed to minimize pain and discomfort to the animals.

### C7 and C8 ventral root avulsion and reimplantation microsurgery

**Figure 6:**
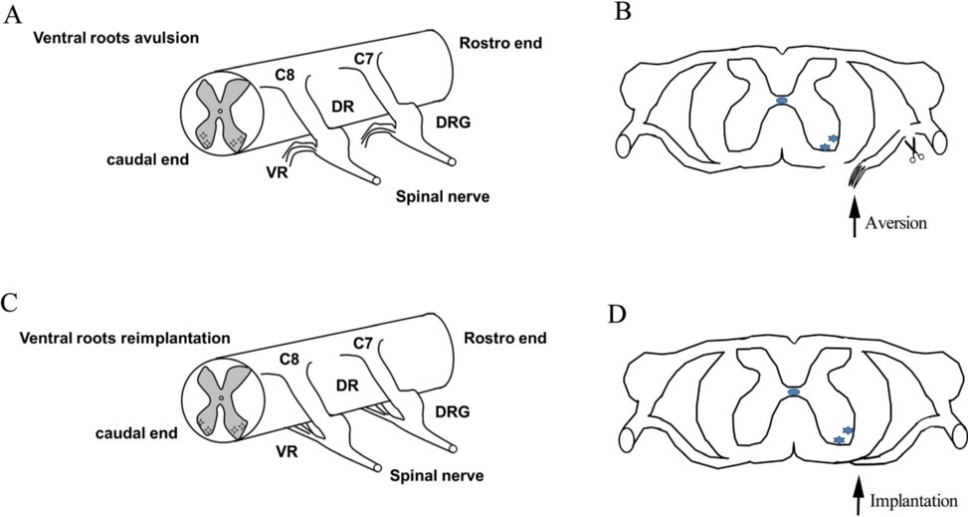
**Schematic drawing of the avulsion and acute reimplantation model.** A unilateral avulsion of the C7-C8 ventral roots **(A)** and implantation of the avulsed C7-C8 ventral root into the ventrolateral surface of spinal cord between the C7 and C8 segments **(C)**. The ventral roots were avulsed and the proximal residual rootlets, as well as the distal parts of the C7 and C8 avulsed roots, were cut away to ensure that spinal MNs would not regrow axons into the C7 and C8 nerves in avulsion rats **(B)**. The avulsed roots were carefully placed and attached onto the ventrolateral surface of spinal cord pia mater **(D)**.

The following procedures were randomly performedon rats: C7 and C8 ventral root avulsion (Av, n = 24 rats) and ventral root avulsion plus immediate reimplantation (Re, n = 24 rats). Avulsion of the right C7 and C8 ventral roots was performed as described in our previous publication [7,68] and further modified in the present study. Briefly, the rat was anesthetized with intraperitoneal injections of 10% chloral hydrate (350 mg/kg). Following the retraction of the paravertebral muscles, the right C6 and C7 laminectomy was performed under a surgical microscope, and then the dura mater was opened. After identifying the C7 and C8 segments of the spinal cord, their ventral roots were selectively avulsed one by one. The proximal residual rootlets and the distal parts of the C7 and C8 avulsed roots were cut away to ensure that spinal MNs would not regrow axons into the C7 and C8 nerves in the avulsed rats. Reimplantation surgery, which was performed to reinsert the avulsed ventral roots into the ventral surface of the corresponding spinal segment, was performed under a microscope immediately after the ventral root avulsion injury in the present study (Figure 6). The procedures of the ventral root reimplantation surgery were performed as previously described [4,11]. Briefly, following C7 and C8 ventral root avulsion, the proximal residual rootlets were retained. Then, the distal parts of the avulsed roots were carefully placed and attached onto the ventrolateral surface of the spinal cord pia mater (Figure 6). More importantly, the reimplanted ventral root was fixed in place with a 10–0 suture (polypropylene) between the cut edges of the dura mater. This procedure also ensured proper cerebrospinal fluid circulation. Finally, in all animals, the muscle, fascia and skin were sutured successively in layers. After the surgery, the animal was returned to its home-cage. All of the manipulation steps were executed by the same technician with the same procedure.

### LiCl treatment

Daily intraperitoneal injection of 1.0 mL phosphate buffer solution (PBS) containing 85 mg/kg LiCl (Sigma, St. Louis, MO, USA) was performed for 12 of the avulsion rats (Av + Li, n = 12) and 12 of the reimplantation rats (Re + Li, n = 12). An injection of 1.0 mL of PBS was used as the control for lithium, which was injected intraperitoneally once a day in 12 of the avulsion rats (Av + PBS, n = 12) and 12 of the reimplantation rats (Re + PBS, n = 12). The duration for rehabilitation was 20 weeks, and none of the animals died during that time.

### Grasping tests

The Grasping tests (GT) were designed to quantify the strength of digit flexion and were described previously [42]. The rat digit flexion of the forepaw was performed by the intrinsic flexor muscles innervated by the median nerve [41]. Briefly, the tail of the rat was gently lifted until only the tested paw grasped a grid connected to an ordinary electronic balance. Then, the rat was lifted further by the tail with the paw firmly grasping the grid. At the moment that the paw lost its grip, the value shown by the electric balance was recorded. Five measurements per forepaw were recorded and the highest value in grams (g) was considered the grasping strength for each rat at each time point. The time interval between each measurement was 5 min. When testing the grasping strength of the ipsilateral paw, the contralateral paws were covered by the adhesive tape before testing.

### Retrograde labeling of regenerating spinal MNs with fluoro-gold

FG retrograde labeling of the spinal MNs was performed following the procedures described in our previous publications [11,69]. Briefly, rats were re-anesthetized at 3 days before the end of the 20 week postinjury period. Under a surgical microscope (Leica, Germany), the right median and ulnar nerves above the cubital fossa were exposed and identified. The injection site was approximately 20 ± 5 mm distal to the nerve cut site of the spinal cord. A total of 1 μl of the FG solution (2% w/v, Fluorochrome, Inc., Englewood, CO, USA) was slowly injected under the epineurium into the proximal stumps of the median and ulnar nerves using a micropipette. The injection lasted for approximately 10 seconds, and then the injection site was clamped with microforceps for another 10 seconds to ensure maximal labeling. Finally, the muscle, fascia and skin were sutured successively in layers. The regenerating axons of the C7 and C8 spinal MNs grew into the reimplanted ventral roots, and the number of FG-labeled MNs was quantified to assess the regenerative capacity of the spinal MNs [11,69]. The FG microinjection through the right median and ulnar nerves of normal rats was used as a control (n = 6) for the FG-labeled MNs.

### Tissue preparation

At the end of the 20 week post-lesion period, all of the animals were administered a lethal dose of chloral hydrate and transcardially perfused with normal saline followed by 4% paraformaldehyde (PFA) in 0.1 M phosphate buffer (PB, pH 7.4). The cervical spinal cord and the brachial plexus were carefully dissected under a microscope to avoid damage to the reimplantation area. The C7 or C8 spinal segments, which were defined as the region between the uppermost and the lowermost roots of the C7 or C8 nerve of the contralateral spinal cord, respectively, together with their dorsal roots and the reimplanted ventral roots were removed. The median nerves, at a location approximately 40 mm distal to the ventral root reimplantation site of the spinal cord, were also harvested. After postfixation by immersion in 4% PFA followed by overnight immersion in 30% (v/v) sucrose in PB solution at 4°C, the transverse sections of the C7 and C8 spinal cord (35 μm), and the median nerves (10 μm) were cut on a cryostat and collected in 0.01 M PBS. Every third section of the spinal cord was used for the investigation of FG-labeled MNs (n = 6) and the ChAT immunofluorescence reaction (n = 6) under a fluorescence microscope. The cross sections of the ventral and dorsal roots were prepared for HE staining (n = 12). Remyelination of the regenerated axons of the MNs was investigated by toluidine blue-stained semithin sections of the median nerves of avulsion (n = 24), reimplantation (n = 24) and normal control (n = 12) animals.

### ChAT immunofluorescence of the spinal cord

The procedures for ChATimmunofluorescencewere similar to those used in our previous study [2]. Briefly, sections were first washed three times with 0.1 M PBS for 10 min and incubated with 0.3% Triton X-100 and 3% bovine serum albumin (BSA) in 0.1 M PBS at room temperature for 30 min. Then, sections were incubated with goat anti-rat ChAT (1:500, Santa Cruz Biotechnology, Dallas, Texas, USA) for 72 h at 4°C. After washing in PBS, the sections were incubated with tetramethylrhodamineisothiocyanate (TRITC)-conjugated rabbit anti-goat IgG (1:200, Sigma, Saint Louis, MO, USA) at room temperature in the dark for 2 h. After washing in PB, sections were mounted on gelatin-coated glass slides and cover slipped in 0.1 M PBS containing 50% glycerin. The ChAT-positive MNs were examined and counted via a fluorescence microscope.

### Counting of ChAT-positive surviving motoneurons

In the slides containing the ChAT immunofluorescence reactions, MNs showing a cytoplasm stained with the ChAT antibody and a visible nucleus were counted on both sides of each C7 and C8 ventral horn under a 20× objective lens. Previous studies have demonstrated that both ipsilateral and contralateral ventral horn MNs are ChAT-positive [23]. Therefore, the number of ChAT-positive MNs on the contralateral side was used as an internal control for each section and expressed as 100%. The number of ChAT-positive MNs in the ipsilateral ventral horn, which indicated the number of surviving MNs, was expressed as a percentage of that of the contralateral ventral horn in the same section. The mean of the number of ChAT-positive MNs of the 6 rats in each subgroup was recorded as the number of the surviving MNs for each subgroup.

### Counting of FG-labeled regenerating motoneurons

Our previous studies have demonstrated that FG labeling through the unilateral median and ulnar nerves was confined to the ipsilateral ventral horn MNs of the C7 and C8 spinal cord segments [70]. Images of the ipsilateral C7 and C8 ventral horns were captured (10 × and 20 × lens) with a Lucida camera attached to a fluorescence microscope (Carl Zeiss, Germany). Counting of the number of the FG-labeled MNs was performed by two people who were blind to rats’ subgroups, and manual counting was performed as described in our previous studies [5,23,71]. The total number of FG-labeled MNs in the ipsilateral C7 and C8 ventral horn were calculated for each rat. The mean of the total number of the 6 rats was recorded as the number of regenerating MNs for each subgroup.

### HE staining of the C7 and C8 spinal roots and toluidine blue staining of semithin sections of the median nerve

The procedure for processing the semithin sections and toluidine blue staining was described in our previous studies [11,72]. Briefly, the median nerves were postfixed in 2.5% glutaraldehyde and subsequently in 1% osmium tetroxide. After dehydrating in a series of graded alcohols, the median nerves were cleared in propylene oxide, and embedded in pure Epon. One millimeter semithin sections were cut on an ultramicrotome (Leica, Germany) and mounted on gelatin-coated glass slides. The myelin of the nerve fibers was stained with 1% toluidine blue. Photographs of the morphology of the spinal cord sections were taken under a light microscope (Carl Zeiss, Germany).

### Counts and measurements of myelinated axons

The images of toluidine blue-stained sections of control, avulsed and implanted median nerves were captured under a light microscope (Carl Zeiss, Germany) and analyzed using Image-Pro Plus 6.0 (Media Cybernetics, MD, USA) software. The method requires the preliminary software procedures of spatial calibration (micron scale) and setting of color segmentation for quantitative color analysis. Sampling bias was avoided by spreading the micrographs systematically over the entire cross-section, according to the procedure proposed by Mayhew and Sharma [73,74]. A total of 3–6 sampling fields in each specimen were selected for counting the total number of myelinated axons. For each axon, the axon diameter was calculated, which was used to determine the distribution of axon diameters.

### Statistics

The statistical calculation and data handling were performed by using SPSS software (version 16.0; SPSS, Chicago, IL). All data are expressed as the mean ± S.E.M. (standard error of the mean). The data obtained in the GT and measurement of BW were analyzed by one-way repeated measures analysis of variance (RM-ANOVA). The test applied to the values from the different time-point assessments followed by Bonferroni post hoc comparisons. The quantification of spinal MNs, including the ChAT immunoreaction (IR), FG retrograde labeling, and median nerve regrowth evaluation by toluidine blue staining, were performed by one-wayANOVA followed by Bonferroni post-hoc comparisons between different treatments. A p value < 0.05 was considered to be statistically significant.

## Abbreviations

Bcl-2: B-cell lymphoma-2; BDNF: Brain-derived neurotrophic factor; BPRA: Brachial plexus root avulsion; BSA: Bovine serum albumin; ChABC: Chondroitinase ABC; ChAT: Choline acetyltransferase; FG: Fluoro-Gold; GSK3: Glycogen synthase kinase-3; MNs: Motoneurons; PB: Phosphate buffer; PBS: Phosphate buffer solution; PFA: Paraformaldehyde.

## Competing interest

The authors have no competing interest with regard to this study.

## Authors’ contributions

All experiments were performed by RF, YT and ZML. YQL, XC and FHS aided in the design of the study. LHZ and WTW conceived of the study and guided the design of experiments. RF and LHZ drafted the manuscript. All authors read and approved the final manuscript.
